# Myasthenic crisis following iodinated contrast material (iohexol) aspiration: a case report

**DOI:** 10.1186/s13256-019-2114-8

**Published:** 2019-05-31

**Authors:** B. V. K. M. Bopeththa, P. B. Hewavithana, H. L. I. Hewapathirana, U. Ralapanawa

**Affiliations:** 1 Emergency Medicine, Teaching Hospital Peradeniya, Peradeniya, Sri Lanka; 2 Radiology Teaching Hospital Peradeniya, Peradeniya, Sri Lanka; 3 Medicine, Teaching Hospital Peradeniya, Peradeniya, Sri Lanka

**Keywords:** Iohexol, Contrast aspiration, Myasthenia gravis, Myasthenic crisis

## Abstract

**Background:**

The number of contrast media-related procedures is ever increasing due to the widespread availability of theoretically safe, low osmolar iodinated contrast material. Although intravenously administered contrast is known to precipitate myasthenic crisis, oral contrast aspiration as a causative factor is not yet documented as such.

**Case summary:**

A 48-year-old Sinhalese man diagnosed as having myasthenia gravis, was evaluated for progressive dysphagia with an upper gastrointestinal contrast study. Iodinated contrast material (iohexol) was used as the contrast medium and there was direct evidence of contrast aspiration during the study. Several minutes after the procedure, severe respiratory distress with evidence of myasthenic crisis requiring intubation and intensive care admission was noted. Treatment with intravenous immunoglobulin, high-dose steroids, and broad-spectrum intravenously administered antibiotics led to an uneventful recovery, although the latter part of the clinical course was complicated with total left lung collapse.

**Summary:**

Myasthenic crisis can be precipitated by various factors and a successful recovery requires mechanical respiratory support with immunomodulatory and steroid therapy. This is the first reported case that describes the development of myasthenic crisis following iohexol-associated aspiration pneumonitis.

## Background

Contrast media-related procedures are considered to be safe in the modern world due to widespread availability of low osmolar contrast media [1]. Allergic reactions, potential nephrotoxicity, and cardiovascular adverse effects are well-known drawbacks to the use of intravenously administered contrast agents. These effects are believed to be minimal with oral contrast use, however, pulmonary aspiration and associated complications are more common with oral contrast use. Moreover, there are only a few elaborative discussions available on adverse effects and mishaps that occurred during oral contrast use [2]. Therefore, we believe that it is worthwhile to report this case which describes how contrast aspiration could precipitate myasthenic crisis. In fact, myasthenia gravis is a rare autoimmune disorder affecting neuromuscular transmission. Myasthenic crisis is a life-threatening complication of myasthenia gravis and it usually requires immunomodulatory therapy and intensive care unit (ICU) admission for ventilatory support. This case report describes a complicated clinical scenario of myasthenic crisis precipitated by aspiration of low osmolar oral contrast material.

## Case presentation

**Fig. 1:**
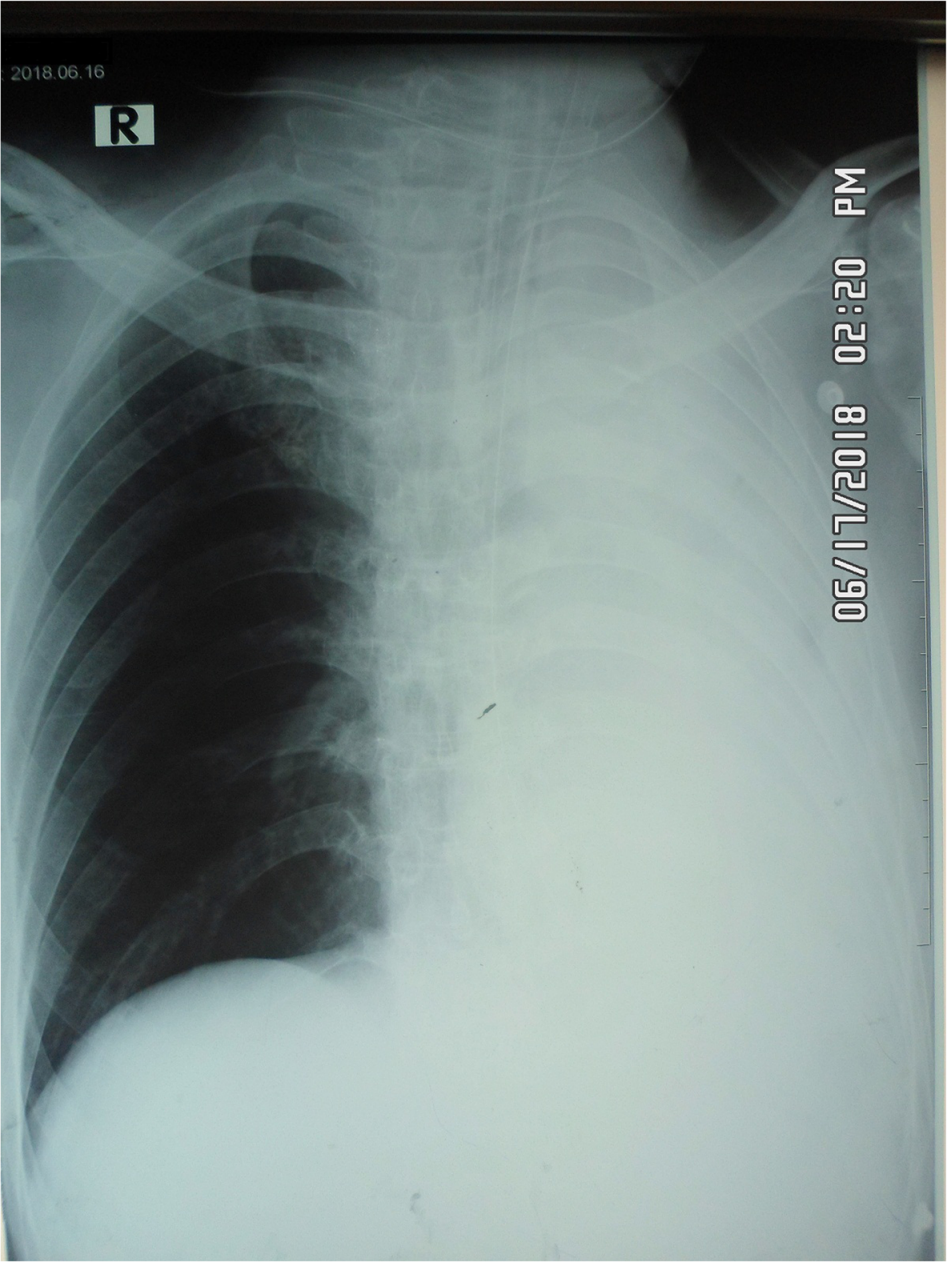
Chest X-ray anteroposterior view showing complete left lung collapse and hyperinflated right lung with right-sided subcutaneous emphysema

A 48-year-old Sinhalese man with myasthenia gravis was presented to the department of radiology of a tertiary care hospital for upper gastrointestinal (GI) contrast study, for further evaluation of progressive dysphagia. Myasthenia gravis was diagnosed in May 2016 and he underwent thymectomy in November 2016 for thymic hyperplasia. From the point of diagnosis, he had two episodes of myasthenic crisis, precipitated by lower respiratory tract infections that required mechanical ventilatory support. Thereafter, he was on regular pyridostigmine, 50 mg/6 hourly, mycophenolate mofetil (MMF) 500 mg twice daily, and orally administered prednisolone therapy. He was able to perform his daily routines of life with negligible support. Meanwhile, he developed progressive dysphagia for solids initially and then for liquids for a 3-month duration. He was evaluated by a neurologist and referred to the surgical team for upper GI endoscopy. Since that was also uneventful, he was referred to our radiology unit for a contrast study. On admission to the radiology unit, he had normal respiratory parameters and his limb muscle power was grade 5/5. Due to the possible risk of aspiration, 10 ml of iohexol (Omnipaque™) was given under fluoroscopy guidance. As the contrast material had directly entered his right main bronchus, the procedure was abandoned and he was transferred to the accident and emergency treatment unit (ETU). Although he was able to maintain his air oxygen saturation above 90% with high flow oxygen via non-rebreather mask, effort of breathing drastically dropped 45 minutes after admission to the ETU including dropping of respiratory rate to 10 breaths per minute. Despite continuous treatment with nebulized salbutamol and intravenously administered metronidazole 500 mg stat dose, he eventually required endotracheal intubation with 3 mg midazolam and 10 mg atracurium administered intravenously. There was a drooping of eyelids, but it was very difficult to assess limb muscle power before intubation. According to the clinical scenario, the diagnosis of respiratory distress due to contrast aspiration was made and he was transferred to the ICU. Following admission, the diagnosis was questioned as there were no significant chest X-ray (CXR) abnormalities to cause the high degree of respiratory distress, except right apical segment collapse. He was then evaluated by a neurologist and the diagnosis was revised to myasthenic crisis, possibly due to aspiration pneumonitis caused by aspiration of contrast. Hence, intravenously administered immunoglobulin 20 g daily for 5 days was commenced with an increased dose of pyridostigmine 60 mg 6 hourly, MMF 750 mg twice daily, and prednisolone 40 mg daily. Although there was no clinical, microbiological, or serological evidence of infection, considering the high possibility of potential development of sepsis, intravenously administered ceftriaxone 1 g 8 hourly was initiated. In addition, there were no electrolyte abnormalities acting as a precipitant of myasthenic crisis. Mechanical ventilation was continued in a synchronized intermittent mandatory mode with fraction of inspired oxygen (FiO_2_) of 50%, positive end-expiratory pressure (PEEP) of 5 cmH_2_O, and pressure support 10 cmH_2_O. Respiratory support was gradually reduced over 72-hour period as there was remarkable improvement in his respiratory mechanics with the treatment. Meanwhile, there was a sudden de-saturation with evidence of subcutaneous emphysema on the right side of his neck and a subsequent CXR revealed complete collapse of his left lung, a hyperinflated right lung with slightly increased translucency on same side, with right-sided subcutaneous air (Fig. 1). CXR findings raised the suspicion of right-sided pneumothorax. Since the cause of subcutaneous emphysema was occult in the CXR, non-contrast computed tomography (NCCT) of his chest was done and revealed total left lung collapse with ipsilateral mediastinal shift and left loculated pneumothorax. Subsequent bronchoscopy was negative. However, with regular chest physiotherapy, lung collapse improved. He was then gradually weaned off the ventilator and transferred to the general medical ward for continuation of care. Following an 18-day hospital stay, he was successfully discharged from the hospital with a tailing off dose of prednisolone. However, due to the high possibility of aspiration, he was advised to keep the nasogastric tube. Two weeks following discharge, during the first clinic visit, he was ambulant showing an improvement in swallowing. The nasogastric tube was removed and regular follow-up was planned.

## Discussion and conclusions

With the evolution of relatively safe non-ionic, low osmolar water-soluble contrast agents such as iohexol (Omnipaque™), the number of contrast fluoroscopic procedures in use is increasing. The osmolality of iohexol ranges from 322 mOsm/kg, which is approximately 1.1 times that of blood plasma, to 844 mOsm/kg, which is almost three times that of blood, whereas hyperosmolar contrast agents have as much as five times the osmolarity of plasma [1, 2]. The assumed safety of iohexol has led to its widespread availability and use. However, only a few studies are available with regard to the direct effects of iohexol on human tissues, especially airways and lungs. According to Wells *et al.*, iohexol is not a completely safe agent on lung tissues [3]. These results were derived from an animal study which was carried out with injections of iohexol and Gastrografin (sodium diatrizoate and meglumine diatrizoate) directly into the trachea of two groups of rats. All of the rats injected with full strength Gastrografin (sodium diatrizoate and meglumine diatrizoate) died immediately due to pulmonary edema, while all the rats injected with iohexol survived. However, peribronchial and microvascular edema and later focal lung collapse were noted in the same group. This was attributed to the osmotic effects of iohexol on lungs. Therefore, this study concluded that iohexol is more of an irritant to the airway than previously believed [3].

According to Harris *et al.*, among 1978 fluoroscopic examinations done in a group of tertiary care centers within a 1-year period, 22% of fluoroscopic examinations had aspirations [4]. However, no adverse effects were noted with the use of iohexol. Therefore, iohexol was concluded a safe low osmolar contrast medium that can safely provide information [4]. So far, no reported deaths have occurred due to iohexol aspiration. Reported deaths were basically due to massive aspiration of barium in elderly debilitated patients. In addition, dysphagia, known neurological diseases, swallowing disorder, and cognitive impairment have been identified as predisposing factors in these deadly conditions. Many of the risk factors mentioned were present in our patient and he was at high risk of aspiration from the beginning of the study.

Myasthenia gravis is a rare autoimmune disorder which has an annual incidence of 0.25–2 patients per 100,000 [5]. The disease is characterized by the presence of defective neuromuscular transmission at the neuromuscular junction. Depending on the serologically present autoimmune antibodies, three types of disease groups can be identified. The most frequently seen group is associated with antibodies against acetylcholine receptors (AChR) in the post-synaptic motor end plate. The second group is associated with antibodies against muscle-specific tyrosine kinase (MuSK). The third group has none of the mentioned antibodies and so is considered seronegative. Myasthenic crisis is a serious complication which is characterized by worsening muscle weakness leading to respiratory failure requiring intubation and mechanical ventilation. Of patients with myasthenia, 15 to 20% can develop this once in their lifetime [6].

In this patient, the initial differential diagnosis for respiratory distress was aspiration pneumonitis, because fluoroscopy images taken during the study showed direct entry of iohexol into his right main bronchus. However, worsening of bilateral ptosis, weak neck muscle power, poor respiratory effort and rate, and negative CXR, refined the diagnosis to myasthenic crisis induced by pulmonary aspiration of contrast material. In this context, Guillain–Barré syndrome, cholinergic crisis, and electrolyte imbalances were also possibilities but rapid onset of symptoms, with underlying history of myasthenia gravis, absence of excess cholinergic symptoms like small pupils or bradycardia, and normal electrolytes respectively, made them remote possibilities. Although there are lists of precipitants of myasthenic crisis, the most common one is infection, which was not evident in this case. The most possible cause in this particular case would be contrast aspiration and procedure-related emotional stress.

The significant threat to life in myasthenic crisis is respiratory failure. Therefore, elective intubation is mandatory after assessing respiratory parameters. The standard 20/30/40 rule (vital capacity < 20 ml/kg; peak inspiratory pressure < 30 cmH_2_O, and peak expiratory pressure < 40 cmH_2_O) is probably the most helpful guide to decide when to intubate [7]. The best choice for a neuromuscular blocking agent for the intubation of such patients is still controversial. Succinylcholine can be safely used in patients with myasthenia gravis. Its most striking feature is the requirement of larger doses of succinylcholine due to the reduced availability of AChR at the post-synaptic cleft. The drawback is the prolonged paralysis [8]. Atracurium has also been used safely in these patients. In contrast to succinylcholine, the dose of atracurium is very much less because of reduced numbers of AChR [9]. Further, atracurium also appears to offer an advantage over other non-depolarizing muscle relaxants in patients with myasthenia gravis, due to its shorter duration and less cumulative effect at the neuromuscular junction [9]. Due to these reasons, atracurium was used to induce muscle paralysis in this patient. However, the main disadvantage of atracurium in rapid sequence induction intubation is a slower onset of action, compared to depolarizing muscle relaxants which are associated with relatively increased risk of aspiration.

The two main pharmacological therapies available for myasthenic crisis are plasma exchange (PE) and intravenous immunoglobulin (IVIg). It is still controversial as to which one is better, due to lack of evidence from properly masked, randomized, standardized trials [10]. As both IVIg and PE have different characteristics, such as rate of onset of clinical efficacy, availability, cost, side effects, and complexity related to the procedure, one can decide on the choice of therapy after considering the above factors. The complexity of the PE procedure, requirement of a central venous line for PE, and rapid availability of IVIg, made IVIg our first choice. Moreover, a relatively rapid recovery also led us to continue the IVIg therapy.

Total left lung collapse and suspicion of right-sided supine pneumothorax also complicated the clinical course of this patient. The presence of right-sided subcutaneous emphysema led us to suspect right-sided supine pneumothorax. The increased translucency and deep sulcus sign on the right side were the suggestive CXR evidence for supine pneumothorax. However, NCCT of his chest revealed a completely different pathology and that was the left-sided loculated pneumothorax. However, as a learning point, the following radiological signs should be considered in the context of supine pneumothorax: increased radiolucency of the affected hemidiaphragm, deep costophrenic sulcus, increased sharpness of cardiac and mediastinal borders, double diaphragmatic sign, and depression of ipsilateral hemidiaphragm [11].

In conclusion, although only a few case reports are available on myasthenic crisis following exposure to intravenously administered contrast, this is the first case reported on myasthenic crisis following iodinated contrast aspiration [12]. Myasthenic crisis is a life-threatening complication which requires prompt mechanical respiratory support and immunomodulatory therapy. Contrast aspiration pneumonitis is the most likely precipitant factor for myasthenic crisis in this patient. Early intubation, vigilance of myasthenic crisis, and comprehensive multidisciplinary intensive care management led to the complete recovery of this patient.

## Abbreviations

AChR: Acetylcholine receptors
CXR: Chest X-ray
ETU: Emergency treatment unit
FiO_2_: Fraction of inspired oxygen
GI: Gastrointestinal
ICU: Intensive care unit
IVIg: Intravenous immunoglobulin
MMF: Mycophenolate mofetil
MuSK: Muscle-specific tyrosine kinase
NCCT: Non-contrast computed tomography
PE: Plasma exchange
PEEP: Positive end-expiratory pressure
